# BMSC exosomes deliver JKAP to restore Th17/Treg balance via AKT/ERK, alleviating rheumatoid arthritis

**DOI:** 10.1016/j.isci.2025.112832

**Published:** 2025-06-06

**Authors:** Fang-Tian Xu, Yu Ling, Hui-Xian Wei, Lingzhang Meng, Dong Yin, Zhong-Hong Lai, Yu Huang, Xiao Huang, Hai-Ye Li, Qin-Wen Luo, Jian Song, Qiang Tang, Hong-Mian Li

**Affiliations:** 1 Department of Orthopedics, The First Affiliated Hospital of Gannan Medical University, Ganzhou, China; 2 Department of Emergency, Guangxi Academy of Medical Sciences & The People’s Hospital of Guangxi Zhuang Autonomous Region, Nanning, China; 3 Key Laboratory of Biomedical Material Research of Guangxi (Cultivation), Affiliated Hospital of Youjiang Medical University for Nationalities, Baise, China; 4 Burn Plastic & Trauma Surgery Department, Affiliated Hospital of Youjiang Medical University for Nationalities, Baise, China; 5 Department of Orthopedics, Guangxi Academy of Medical Sciences & The People’s Hospital of Guangxi Zhuang Autonomous Region, Nanning, China; 6 Institute of Cardiovascular Medicine, Guangxi Academy of Medical Sciences & The Guangxi Academy of Medical Sciences, Nanning, China; 7 Research Center of Medical Sciences, Department of Plastic and Aesthetic Surgery, Guangxi Academy of Medical Sciences & The People’s Hospital of Guangxi Zhuang Autonomous Region & Research Center of Medical Sciences, Nanning, China; 8 Department of Orthopedics, The Ninth People's Hospital of Nanning, Binyang, China; 9 Department of Plastic and Aesthetic Surgery & Research Center of Medical Sciences, Guangxi Academy of Medical Sciences & The People’s Hospital of Guangxi Zhuang Autonomous Region, Nanning, China

**Keywords:** Immunology, Immunity, Cell biology

## Abstract

JKAP regulates T cell immunity and inflammation in autoimmune diseases. This study investigates how bone marrow mesenchymal stem cell (BMSC)-derived exosomes deliver JKAP to restore Th17/Treg balance in rheumatoid arthritis (RA). RA CD4^+^ T-cells and fibroblast-like synoviocytes (RA-FLS) were treated with BMSC, exosomes, or JKAP-modified exosomes ± AKT/ERK inhibitors. BMSC/exosomes suppressed Th17 and promoted Treg differentiation, effects diminished without exosomes. JKAP knockdown impaired exosome-mediated Th17/Treg regulation and AKT/ERK activation, while overexpression enhanced these effects. JKAP-deficient exosomes increased CD4^+^ T cell proliferation and RA-FLS inflammation, reversed by AKT/ERK inhibition. In collagen-induced arthritis mice, exosomes alleviated symptoms and restored Th17/Treg balance, whereas JKAP knockdown exacerbated arthritis and disrupted balance. JKAP-overexpressing exosomes showed opposite effects. Thus, BMSC exosomes mitigate RA by delivering JKAP to restore immune balance via AKT/ERK pathways.

## Highlights

• BMSC exosomes deliver JKAP to restore Th17/Treg balance in rheumatoid arthritis
• JKAP suppresses AKT/ERK activation, reversing Th17-driven inflammation
• JKAP-enriched exosomes alleviate arthritis severity in a mouse model
• Exosomal JKAP inhibits synovial cell proliferation and inflammatory cytokine release

## Graphical abstract

**Figure:**
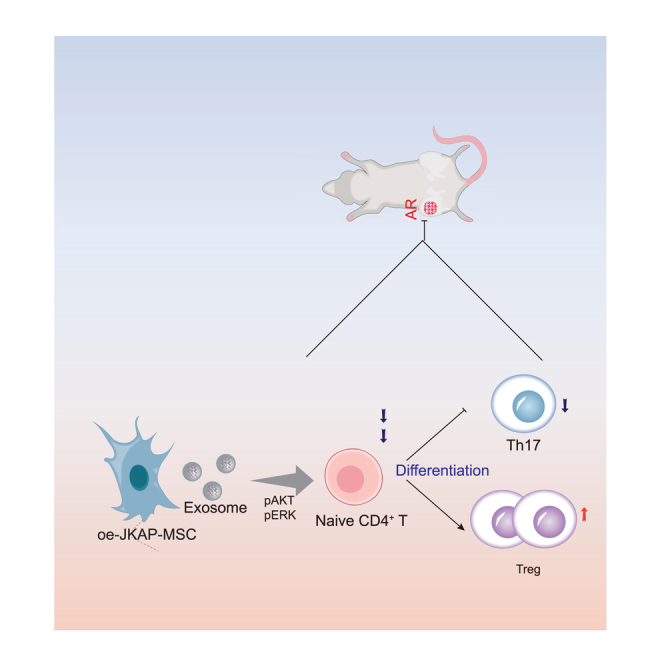

## Introduction

Rheumatoid arthritis (RA) is a chronic autoimmune disease predominantly affecting middle-aged and older women.^1^^,^^2^ Despite advances in novel drugs and management strategies, patients still experience low remission rates and high flare risks.^3^^,^^4^^,^^5^ Cell therapy, an innovative treatment option, shows potential in treating autoimmune diseases like systemic lupus erythematosus, psoriasis, and RA.^6^^,^^7^^,^^8^ Among various cell therapies, bone marrow mesenchymal stem cell (BMSC) therapy is pivotal. Beyond its pluripotent differentiation and anti-inflammatory effects, BMSC therapy may treat autoimmune diseases, including RA, through exosome delivery.^9^^,^^10^ However, the mechanisms remain complex and not fully understood.^11^^,^^12^^,^^13^

JNK pathway-associated phosphatase (JKAP) is a protein tyrosine phosphatase involved in T cell mediated immunity and inflammation.^14^^,^^15^ It has been linked to reduced progression of autoimmune diseases such as juvenile idiopathic arthritis and inflammatory bowel disease by influencing T helper (Th) cell differentiation.^16^^,^^17^^,^^18^ In RA, JKAP is associated with decreased inflammation, synovitis, and disease activity. Increased JKAP levels during treatment correlate with positive responses to tumor necrosis factor inhibitors or conventional disease-modifying anti-rheumatic drugs.^19^^,^^20^^,^^21^

Previous studies have suggested that JKAP plays a crucial role in regulating immune responses by modulating T cell activity through the JNK signaling pathway.^22^ It acts as a negative regulator to suppress excessive immune responses. In RA, dysregulation of JKAP can lead to an imbalance between Th17 and Treg cells, resulting in increased inflammation and autoimmunity.^21^ Th17 cells are pro-inflammatory,^23^ whereas Treg cells are anti-inflammatory and help maintain immune tolerance. A shift toward Th17 dominance over Treg cells in RA exacerbates disease progression. JKAP’s influence on cytokine production and T cell differentiation links it to this balance,^24^ suggesting it as a potential therapeutic target to restore the Th17/Treg balance, thereby reducing inflammation and joint damage. The above information indicates the close involvement of JKAP and its potency as treatment option for RA. The current study aimed to investigate the implication of JKAP in the BMSC treatment on RA, as well as its interaction with Th17/Treg balance and the underlying mechanisms.

## Results

### BMSC attenuates Th17/Treg imbalance via exosomes in RA CD4^+^ T cells

Bone marrow-derived mesenchymal stem cells (BMSCs) were isolated, and their morphology was observed (Figure S1A). Marker analysis revealed high levels of CD29, CD44, CD90, and CD105, with low levels of CD34 and CD45 (Figure S1B). BMSC-derived exosomes were isolated, predominantly measuring 50–150 nm in size (Figure S1C), and exhibited high expression of CD63, CD81, and TSG101 (Figure S1D).

Interestingly, JKAP expression was found to be downregulated in RA BMSCs compared to control BMSCs (Figure S1E), as well as in RA BMSC-derived exosomes compared to control BMSC-derived exosomes (Figure S1F).

**Figure 1:**
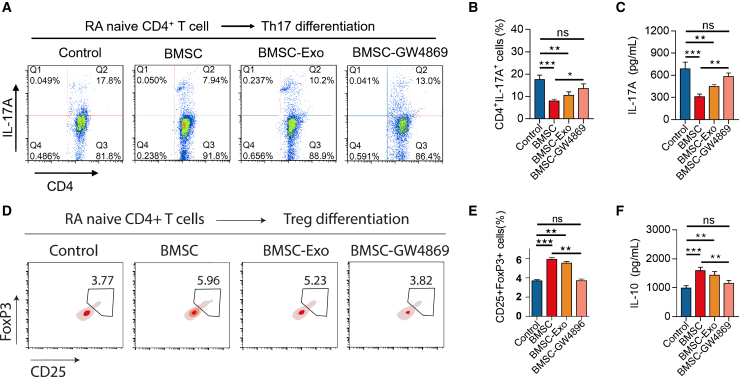
Effect of BMSC and its exosome on RA Th17/Treg differentiation (A–F) CD4^+^IL- 17A^+^ cell percentage (A-B) and IL-17A level (C) among groups. CD25^+^Foxp3^+^ cell percentage (D-E) and IL-10 level (F) among groups. Data are represented as mean ± SEM. 8 samples in each group. Similar results were acquired from at least 3 independent experiments. ns: no significance; ∗: *p* < 0.05; ∗∗: *p* < 0.01; ∗∗∗: *p* < 0.001.

Both BMSCs and their exosomes significantly suppressed the percentage of RA CD4+IL-17A + cells and the level of IL-17A (all *p* < 0.01). In contrast, BMSCs without exosomes (treated with GW4869) had a reduced impact on these levels (both *p* > 0.05) (Figures 1A–1C). Additionally, both BMSCs and their exosomes promoted the percentage of RA CD25+Foxp3+ cells and the level of IL-10 (all *p* < 0.05), whereas BMSCs without exosomes showed less effect (both *p* > 0.05) (Figures 1D–1F). These findings suggest that BMSCs attenuate the Th17/Treg imbalance in RA via their exosomes.

### JKAP modifies BMSC-Exosome’s effect on Th17/Treg differentiation and AKT/ERK activation in RA CD4^+^ T cells

**Figure 2:**
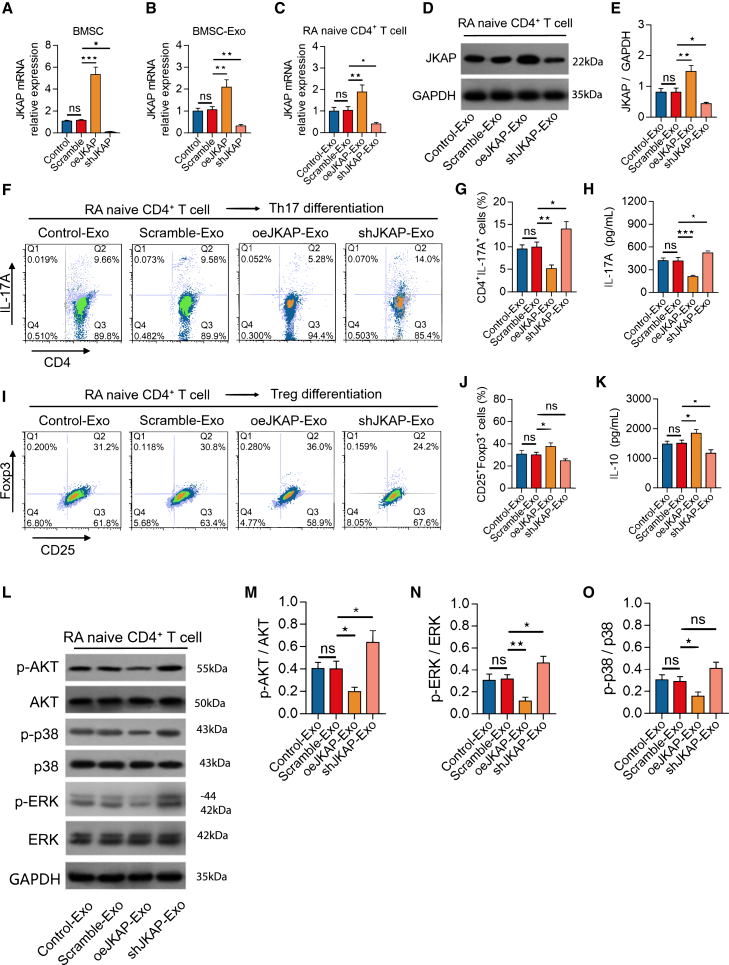
Effect of JKAP-modified BMSC-exosome on RA Th17/Treg differentiation (A–O) JKAP mRNA expression of BMSC (A) and its exosome (B) among groups. JKAP mRNA expression (C) and protein expression (D-E) in RA CD4^+^ T cells among groups. CD4^+^IL- 17A^+^ cell percentage (F-G) and IL-17A level (H) among groups. CD25^+^Foxp3^+^ cell percentage (I-J) and IL-10 level (K) among groups. (L) Quantification of the expression of *p*-AKT (M), *p*-ERK (N) and p-p38 (O). Data are represented as mean ± SEM. 6 samples in each group. Similar results were acquired from at least 5 independent experiments. ns: no significance; ∗: *p* < 0.05; ∗∗: *p* < 0.01; ∗∗∗: *p* < 0.001.

Following transfection, JKAP expression was decreased in BMSCs and their exosomes transfected with shJKAP lentivirus compared to those with Scramble lentivirus (both *p* < 0.05). Conversely, JKAP expression increased in BMSCs and their exosomes transfected with oeJKAP lentivirus compared to Scramble lentivirus (both *p* < 0.01) (Figures 2A and 2B). After treatment with exosomes, JKAP-knockdown BMSC-exosomes reduced JKAP expression in RA CD4^+^ T cells compared to Scramble BMSC-exosomes (*p* < 0.05), while JKAP-overexpression BMSC-exosomes elevated it (*p* < 0.01) (Figures 2C–2E).

JKAP-knockdown BMSC-exosomes increased the percentage of RA CD4+IL-17A + cells (*p* < 0.05) and the level of IL-17A (*p* < 0.05) (Figures 2F–2H), but did not affect the percentage of RA CD25+Foxp3+ cells (*p* > 0.05) and inhibited the level of IL-10 (*p* < 0.05) compared to Scramble BMSC-exosomes (Figures 2I–2K). It has been reported that phosphorylated AKT (*p*-AKT), phosphorylated ERK (*p*-ERK), and phosphorylated p38 (p-p38) are involved in the regulation of Th17/Treg balance.^25^^,^^26^^,^^27^^,^^28^ In this study, JKAP-knockdown BMSC-exosomes increased the expression of phosphorylated AKT (*p*-AKT) (*p* < 0.05) (Figures 2L and 2M) and phosphorylated ERK (*p*-ERK) (*p* < 0.05) (Figures 2L and 2N) in RA CD4^+^ T cells, but did not affect the expression of phosphorylated p38 (p-p38) (*p* > 0.05) (Figures 2L and 2O), compared to Scramble BMSC-exosomes. Conversely, JKAP-overexpression BMSC-exosomes exhibited the opposite effects on these markers (all *p* < 0.05). These findings suggest that JKAP plays a critical role in the function of BMSC-exosomes in regulating Th17/Treg imbalance and the activation of AKT and ERK in RA CD4^+^ T cells.

### JKAP-modified BMSC-Exosomes regulate CD4^+^ T cells to influence RA-FLS growth, invasion, and inflammation

**Figure 3:**
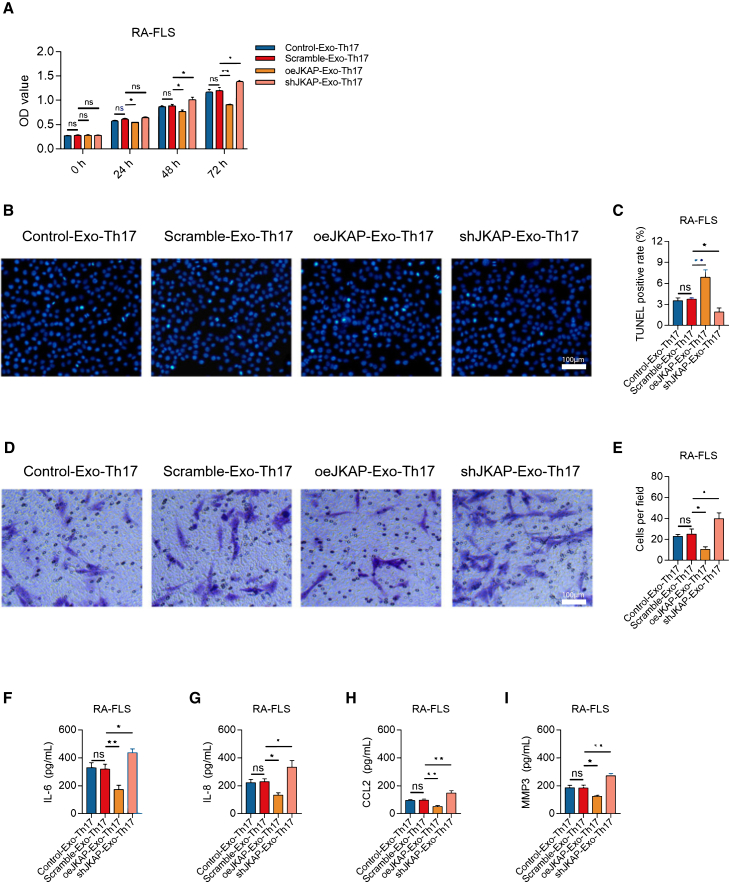
Effect of JKAP modified BMSC-exosome stimulating CD4^+^ T cells on RA-FLS growth, invasion and inflammation (A–I) RA-FLS proliferation (A), TUNEL image examples (B), quantified apoptosis rate by TUNEL (C), Transwell image examples (D) among groups, quantified cell invasion by Transwell (E), Levels of IL-6 (F), IL-8 (G), CCL2 (H), MMP3 (I) among groups in RA-FLS supernatant. Data are represented as mean ± SEM. 8 samples in each group. Similar results were acquired from at least 2 independent experiments. For (B), it was captured at 5x lens, for (D), it was captured at 20x lens. ns: no significance; ∗: *p* < 0.05; ∗∗: *p* < 0.01.

CD4^+^ T cells, after co-culture with JKAP-modified BMSC-exosomes and Th17 polarization, were used to treat RA fibroblast-like synoviocytes (RA-FLS). Compared to Scramble BMSC-exosomes, JKAP-knockdown BMSC-exosomes promoted RA-FLS proliferation (*p* < 0.05) (Figure 3A), suppressed apoptosis (*p* < 0.05) (Figures 3B and 3C), and facilitated invasion (*p* < 0.05) (Figures 3D and 3E). Additionally, JKAP-knockdown BMSC-exosomes increased the release of inflammatory cytokines, including IL-6 (*p* < 0.05) (Figure 3F), IL-8 (*p* < 0.05) (Figure 3G), CCL2 (*p* < 0.01) (Figure 3H), and MMP3 (*p* < 0.01) (Figure 3I). Conversely, JKAP-overexpression BMSC-exosomes exhibited opposite effects on RA-FLS growth, invasion, and inflammation (all *p* < 0.05). These results indicate that JKAP-modified BMSC-exosomes regulate Th17 differentiation, thereby affecting RA-FLS growth, invasion, and inflammation.

### Effects of MK2206 and PD98059 treatment along with JKAP knockdown on RA Th17 differentiation and RA-FLS functions

**Figure 4:**
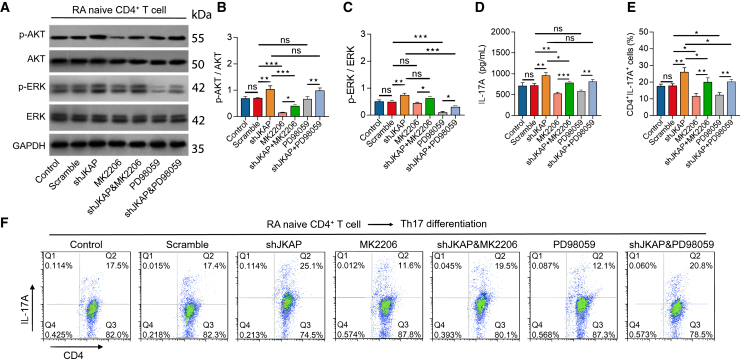
Direct JKAP knockdown, MK2206 and PD98059 treatment on RA Th17 differentiation (A–F) Western blot image examples (A), quantified *p*-AKT expression (B), quantified *p*-ERK expression (C) in RA CD4^+^ T cells among groups. IL-17A level (D) and CD4^+^IL-17A^+^ cell percentage (E-F) among groups. Data are represented as mean ± SEM. 6 samples in each group. Similar results were acquired from at least 3 independent experiments. ns: no significance; ∗: *p* < 0.05; ∗∗: *p* < 0.01; ∗∗∗: *p* < 0.001.

Direct knockdown of JKAP using lentivirus decreased JKAP expression (*p* < 0.01) (Figures S2A–S2C) and increased the expression of phosphorylated AKT (*p*-AKT) (*p* < 0.01) (Figures 4A and 4B) and phosphorylated ERK (*p*-ERK) (*p* < 0.01) (Figures 4A and 4C) in RA CD4^+^ T cells. MK2206 treatment suppressed *p*-AKT (*p* < 0.001) and PD98059 treatment suppressed *p*-ERK (*p* < 0.001) in RA CD4^+^ T cells, irrespective of JKAP knockdown (Figures 4A–4C).

**Figure 5:**
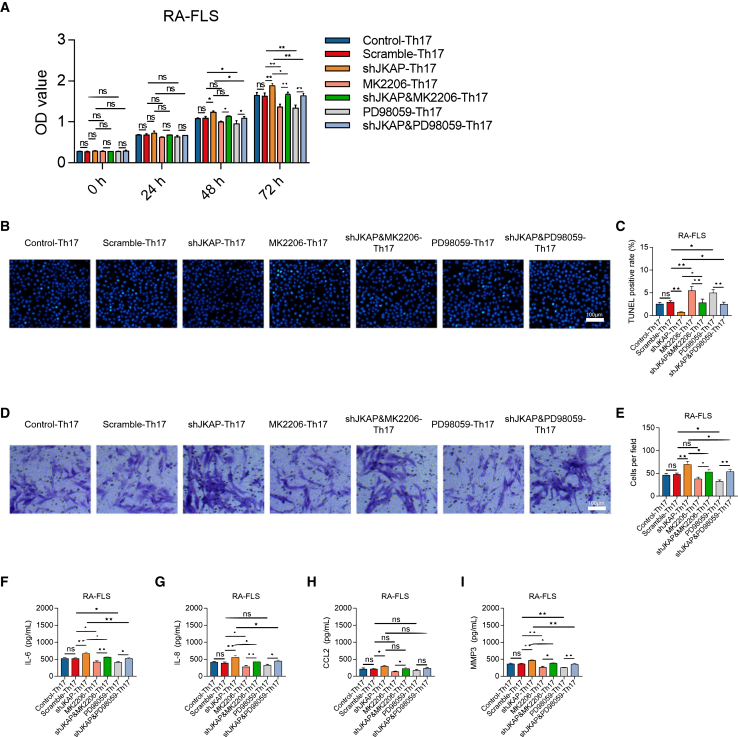
JKAP knockdown/MK2206/PD98059 stimulating CD4^+^ T cells on RA-FLS growth, invasion and inflammation (A–I) RA-FLS proliferation (A), TUNEL image examples (B), quantified apoptosis rate by TUNEL (C), Transwell image examples (D) among groups), quantified cell invasion by Transwell (E). Levels of IL-6 (F), IL-8 (G), CCL2, (H), MMP3 (I) among groups in RA-FLS supernatant. Data are represented as mean ± SEM. 10 samples in each group. Similar results were acquired from at least 3 independent experiments. For (B), it was captured at 5x lens, for (D), it was captured as 20x lens. ns: no significance; ∗: *p* < 0.05; ∗∗: *p* < 0.01.

JKAP knockdown enhanced the potency of CD4+IL-17A + cells (*p* < 0.01) and increased IL-17A levels (*p* < 0.01), effects that were mitigated by both MK2206 and PD98059 treatments (all *p* < 0.05) (Figures 4D–4F). Furthermore, Th17 polarization was performed in JKAP-knockdown CD4^+^ T cells, which were then used to treat RA fibroblast-like synoviocytes (RA-FLS). JKAP knockdown in Th17 cells increased RA-FLS proliferation (*p* < 0.01) (Figure 5A), reduced apoptosis (*p* < 0.01) (Figures 5B and 5D), enhanced invasion (*p* < 0.01) (Figures 5C and 5E), and stimulated the release of inflammatory cytokines, including IL-6 (*p* < 0.01) (Figure 5F), IL-8 (*p* < 0.01) (Figure 5G), CCL2 (*p* < 0.05) (Figure 5H), and MMP3 (*p* < 0.01) (Figure 5I). Notably, these effects of JKAP knockdown were attenuated by both MK2206 and PD98059 treatments (all *p* < 0.05). These findings imply that JKAP knockdown promotes Th17 cell differentiation via the AKT and ERK pathways, facilitating RA-FLS growth, invasion, and inflammation.

### JKAP-modified BMSC-exosome treatment in CIA animal model

**Figure 6:**
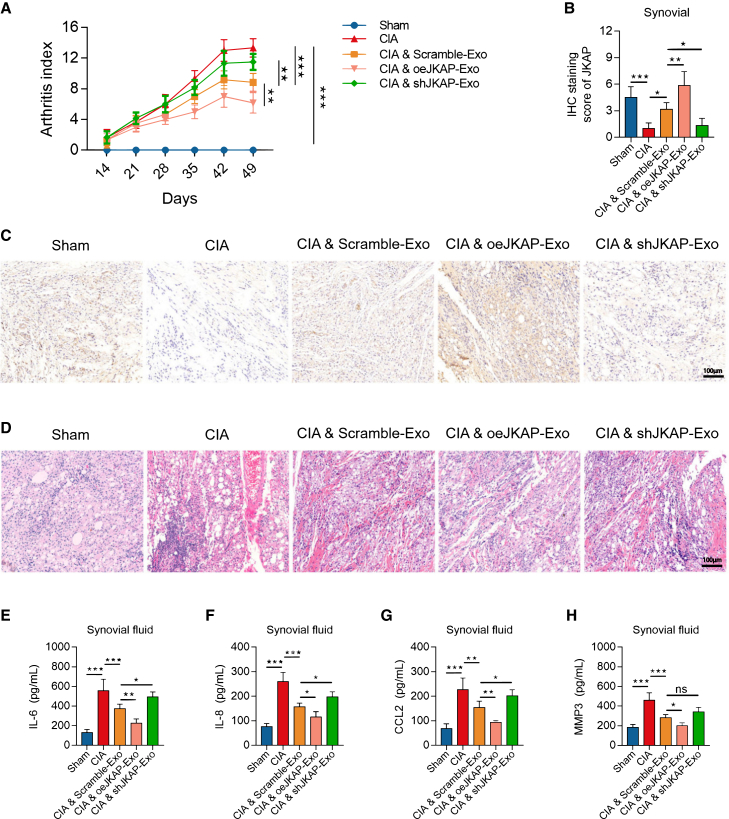
Effect of JKAP modified BMSC-exosome on function, synovial hyperplasia and inflammation in CIA model (A–H) Arthritis index (A), quantified expression of JKAP in synovium (B), IHC image examples of JKAP in synovium (C), HE staining image examples of synovium (D) among groups. Levels of IL-6 (E), IL-8 (F), CCL2 (G), MMP3 (H) in synovial fluid among groups. Data are represented as mean ± SEM. 6 samples in each group. Similar results were acquired from at least 3 independent experiments. For (C), it was captured at 5x lens, for (D), it was captured at 5x lens. ns: no significance; ∗: *p* < 0.05; ∗∗: *p* < 0.01; ∗∗∗: *p* < 0.001.

**Figure 7:**
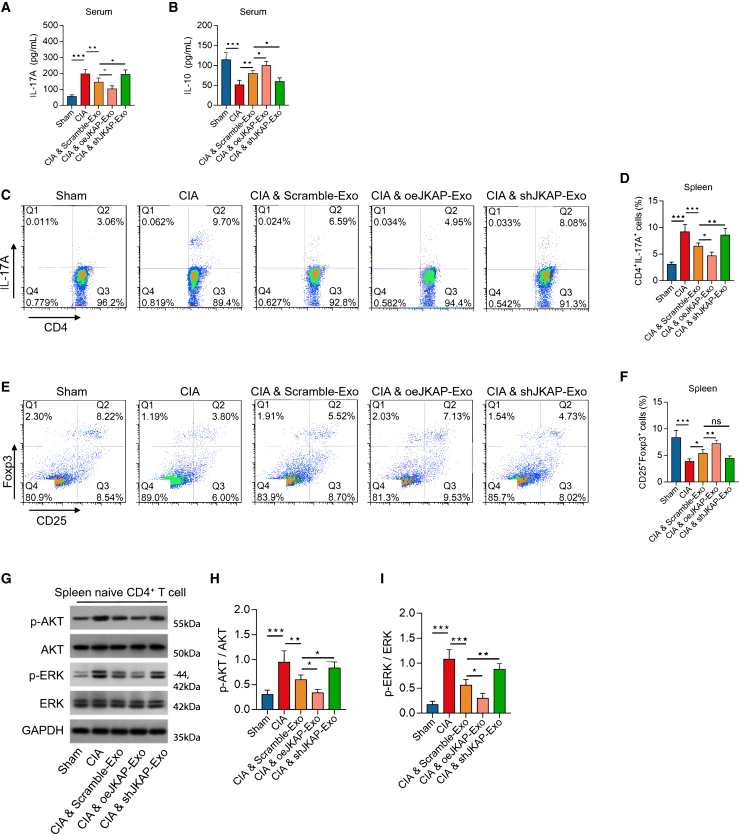
Effect of JKAP modified BMSC-exosome on Th17/Treg imbalance, AKT and ERK activation in CIA model (A–I) Serum IL-17A (A), serum IL-10 (B), spleen CD4^+^IL-17A^+^ cell percentage (C, D), spleen CD25^+^Foxp3^+^ cell percentage (E, F) among groups. Western blot image examples (G), quantified *p*-AKT expression (H), quantified *p*-ERK expression. (I) in spleen CD4^+^ T cells among groups. Data are represented as mean ± SEM. 8 samples in each group. Similar results were acquired from at least 3 independent experiments. ns: no significance; ∗: *p* < 0.05; ∗∗: *p* < 0.01; ∗∗∗: *p* < 0.001.

In this study, compared to the CIA model with no treatment, CIA mice treated with Scramble BMSC-exosomes showed improvements in arthritis index, synovial hyperplasia, and inflammation (all *p* < 0.05), along with a decreased percentage of Th17 cells and an increased percentage of Treg cells in blood and spleen (all *p* < 0.05) (Figures 6A–6H and 7A–7I).

Compared to Scramble BMSC-exosomes, JKAP-knockdown BMSC-exosomes increased the arthritis index (*p* < 0.01) (Figure 6A), reduced JKAP expression (*p* < 0.05) (Figures 6B and 6C), increased synovial hyperplasia and inflammatory infiltration (Figure 6D), and elevated levels of inflammatory cytokines in synovial fluid, including IL-6 (*p* < 0.05) (Figure 6E), IL-8 (*p* < 0.05) (Figure 6F), and CCL2 (*p* < 0.05) (Figure 6G), but not MMP3 (*p* > 0.05) (Figure 6H). Conversely, JKAP-overexpression BMSC-exosomes exhibited the opposite effects on these parameters.

Additionally, compared to Scramble BMSC-exosomes, JKAP-knockdown BMSC-exosomes increased serum IL-17A levels (*p* < 0.05) (Figure 7A), decreased serum IL-10 levels (*p* < 0.05) (Figure 7B), elevated the percentage of spleen CD4+IL-17A + cells (*p* < 0.01) (Figures 7C and 7D), had less effect on the percentage of spleen CD25+Foxp3+ cells (*p* > 0.05) (Figures 7E and 7F), and facilitated the expression of *p*-AKT (*p* < 0.05) and *p*-ERK (*p* < 0.01) in spleen CD4^+^ T cells (Figures 7G–7I) in CIA mice. In contrast, JKAP-overexpression BMSC-exosomes showed opposite effects on these indices. These findings validate that BMSC-exosomes attenuate synovial hyperplasia, inflammation, and symptoms by delivering JKAP to restore the Th17/Treg balance in RA. A hypothesis diagram summarizing the main findings of this study is presented in Figure S3.

## Discussion

The findings of this study elucidate the pivotal role of JKAP in mediating the effects of BMSC-derived exosomes on the Th17/Treg balance in RA. Previous research has identified JKAP as a crucial regulator in various autoimmune diseases, particularly in RA, where its downregulation correlates with disease severity and poor treatment response.^20^^,^^29^ However, the precise mechanisms through which JKAP influences T cell differentiation and the inflammatory milieu in RA have remained poorly understood. This study addresses this gap by demonstrating that BMSC-derived exosomes can deliver JKAP to naive CD4^+^ T cells, promoting Treg differentiation while inhibiting Th17 cell polarization.

Exosomes are known to carry bioactive molecules, including proteins and microRNAs, which play significant roles in intercellular communication.^30^ Our study builds on previous findings that highlighted the role of BMSC-derived exosomes in modulating immune responses in RA.^31^ Notably, we provide novel evidence that JKAP is significantly downregulated in RA BMSCs and their exosomes compared to controls, suggesting that the loss of JKAP may contribute to the Th17/Treg imbalance observed in RA patients. This aligns with earlier studies indicating that microRNAs from BMSC exosomes can influence RA pathogenesis.^32^ Our findings extend this knowledge by implicating JKAP as a key component of the anti-inflammatory properties of BMSC exosomes.

The advantages of this study lie in its comprehensive approach to understanding the cellular mechanisms underlying JKAP’s role in RA. By utilizing both *in vitro* and *in vivo* models, we demonstrate that the modulation of JKAP levels within BMSC-derived exosomes can significantly affect CD4^+^ T cell behavior and RA fibroblast-like synoviocyte (RA-FLS) function. Moreover, our results indicate that JKAP influences critical signaling pathways, including AKT and ERK, which are known to regulate T cell activation and differentiation.^33^ This multifaceted analysis not only corroborates existing literature but also introduces a novel therapeutic perspective, suggesting that enhancing JKAP expression in BMSC-derived exosomes could serve as a potential strategy for restoring immune balance in RA.

Despite these contributions, this study has limitations that warrant consideration. First, while we have established a causal relationship between JKAP and the Th17/Treg balance, further mechanistic studies are needed to explore the downstream effects of JKAP signaling in greater detail. Additionally, while our findings in the CIA mouse model provide valuable insights into the *in vivo* relevance of our observations, the translation of these results to human RA remains to be fully validated. Future studies should aim to investigate the therapeutic potential of JKAP-modified exosomes in clinical settings, assessing their safety and efficacy in human subjects.

In conclusion, our study provides compelling evidence that JKAP plays a critical role in the immune regulatory functions of BMSC-derived exosomes, offering new insights into the pathophysiology of RA. By enhancing our understanding of JKAP’s mechanisms, we pave the way for novel therapeutic approaches aimed at restoring immune homeostasis in autoimmune diseases.

### Limitations of this study

While this study demonstrates that BMSC-derived exosomes alleviate RA by delivering JKAP to restore Th17/Treg balance via AKT/ERK pathways, several limitations warrant consideration. First, the translational relevance of findings from the collagen-induced arthritis (CIA) mouse model to human RA requires further validation, as murine immune responses and disease progression may not fully mirror human pathophysiology. Second, the mechanistic focus on AKT and ERK pathways, while critical, does not exclude potential contributions from other signaling cascades, such as JNK or p38 MAPK, which were not systematically explored. Third, exosome isolation relied on a single commercial kit, and variations in isolation methods (e.g., ultracentrifugation vs. polymer-based precipitation) may influence exosome purity and functional outcomes. Additionally, the study utilized RA-FLS isolated from a single patient, limiting generalizability to the broader RA population. While JKAP knockdown/overexpression experiments clarified its role, the regulatory mechanisms governing JKAP expression in BMSCs and exosomes remain undefined. Finally, long-term safety and off-target effects of JKAP-modified exosomes were not assessed, and the small sample size in the CIA model (*n* = 6 per group) may reduce statistical robustness. Future studies should address these gaps through multi-center human trials, broader pathway analyses, and extended safety evaluations.

This study highlights the therapeutic potential of bone marrow-derived mesenchymal stem cell (BMSC) exosomes in RA by restoring Th17/Treg balance through JKAP modulation. BMSC exosomes effectively reduced pro-inflammatory CD4+IL-17A + cells and IL-17A levels while enhancing anti-inflammatory CD25+Foxp3+ cells and IL-10 levels. The modulation of JKAP in these exosomes was crucial, influencing AKT and ERK signaling pathways that govern Th17/Treg differentiation.

*In vivo*, JKAP-modified BMSC exosomes alleviated synovial hyperplasia, reduced inflammation, and improved arthritis symptoms in a CIA mouse model. These findings underscore the potential of BMSC exosomes as a novel therapeutic approach for RA, offering targeted immune modulation and inflammation reduction. Further research should aim to translate these promising results into clinical applications for RA treatment.

## Acknowledgments

This work was financially supported by the 10.13039/501100001809National Nature Science Foundation of China (82260433), the Guangxi Natural Science Foundation (2023GXNSFDA026035) and the Central Government Guided Local Development of Science And Technology Program (Nanning, 20233013).

## Author contributions

H.-M.L., L.-Z.M., and J.S. designed this study. F.-T.X., Y.L., H.-X.W., and Q.T. perform the animal experiments and most of *in vitro* study. D.Y., Z.-H.L., Y.H., H.-Y.L., and Q.-W.L. helped perform animal experiments and some cell culture experiments.

## Declaration of interests

The authors declare that they have no conflicts of interest.

## STAR★Methods

### Key resources table

**Table:** 

REAGENT or RESOURCE	SOURCE	IDENTIFIER
**Antibodies**		

CD29 antibody	Abcam, USA	ab52971; RRID:AB_870695
CD34 antibody	Abcam, USA	ab81289; RRID:AB_1640332
CD44 antibody	Abcam, USA	ab157107; RRID:AB_2847847
CD45 antibody	Abcam, USA	ab10558; RRID:AB_442947
CD90 antibody	Abcam, USA	ab225; RRID:AB_2201388
CD105 antibody	Abcam, USA	ab11415; RRID:AB_298028
CD63 antibody	Affinity, China	AF1474; RRID:AB_2832907
CD81 antibody	Affinity, China	AF5108; RRID:AB_2837592
TSG101 antibody	Affinity, China	AF8258; RRID:AB_2845322
JKAP antibody	Invitrogen, USA	PA5-103927; RRID:AB_2853179
AKT antibody	Affinity, China	AF6261; RRID:AB_2835121
p-AKT antibody	Affinity, China	AF0016; RRID:AB_2810278
p38 antibody	Affinity, China	AF6456; RRID:AB_2835135
p-p38 antibody	Affinity, China	AF4001DM; RRID:AB_2835330
ERK antibody	Affinity, China	AF0155; RRID:AB_2833329
p-ERK antibody	Affinity, China	AF1015; RRID:AB_2834432
JNK antibody	Affinity, China	AF6318; RRID:AB_2835178
p-JNK antibody	Affinity, China	AF3318; RRID:AB_2834752
GAPDH antibody	Invitrogen, USA	MA5-15738; RRID:AB_10977387
Anti-IL-4 antibody	Affinity, China	DF7557; RRID:AB_2841062
Anti-IFN-γ antibody	Affinity, China	AF5125; RRID:AB_2837609
HRP-linked secondary antibody	Invitrogen, USA	G-21040; RRID:AB_2536527

**Chemicals, peptides, and recombinant proteins**		

GW4869	Selleck, China	S7609
TGF-β	Sigma, USA	T7039
IL-6	Sigma, USA	I1395
IL-1β	Peprotech, USA	200-01B
IL-23	Peprotech, USA	200-23
IL-2	Peprotech, USA	200-02
MK2206	Selleck, USA	S1078
PD98059	Selleck, USA	S1177
Polybrene	Beyotime, China	C0351
Chicken type II collagen	Chondrex, USA	20011
Complete Freund’s adjuvant	Chondrex, USA	7001
PMA	BD, USA	555598
Ionomycin	BD, USA	560821
BD GolgiStop™	BD, USA	554724

**Critical commercial assays**		

Hieff® Quick Exosome Isolation Kit	Yeasen, China	41201ES50
BCA Protein Assay Kit	Sangon, China	C503051
Human CD4 Naïve T Cell Isolation Kit	Stemcell, USA	19555
T-cell Culture and Expansion Medium	Takara, Japan	TCH-C100
Th17/Treg Phenotyping Kit (Human)	BD, USA	560755
Th17/Treg Phenotyping Kit (Mouse)	BD, USA	560756
Total Exosome RNA & Protein Isolation Kit	Invitrogen, USA	4478545
Trizol Reagent	Sangon, China	B610409
First Strand cDNA Synthesis Kit	Beyotime, China	D7168L
SYBR Green qPCR Master Mix	Beyotime, China	D7260
Cell Counting Kit-8	GlpBio, USA	GK10001
TUNEL Assay Kit	Beyotime, China	C1088
HE Stain Kit	Solarbio, China	G1120
DAB Kit	Solarbio, China	DA1010
ELISA Kits (IL-17A, IL-10, IL-6, IL-8, CCL2, MMP3)	Solarbio, China	SEKH-0013, SEKH-0010, SEKH-0006, SEKH-0008, SEKH-0028, SEKM-0024
Mouse CD4^+^ T Cell Isolation Kit	Stemcell, USA	19852
Mouse CD4 Naïve T Cell Enrichment Kit	NovoBio, China	NBP2-50151

**Experimental models: cell lines**		

Normal human BMSC	Procell Life Science & Technology, China	CVCL_A9JT; RRID:CVCL_A9JT

**Experimental models: organisms/strains**		

Mouse: Female DBA/1	Local supplier (not specified)	N/A

**Recombinant DNA**		

Lentivirus: oeJKAP	Hanbio Biotechnology, China	Custom
Lentivirus: shJKAP	Hanbio Biotechnology, China	Custom
Lentivirus: Scramble	Hanbio Biotechnology, China	Custom

**Software and algorithms**		

GraphPad Prism 7	GraphPad, USA	Version 7; RRID:SCR_002798
Image J	NIH, USA	Version 1.8; RRID:SCR_003070
Nanosight NS300 Software	Malvern, UK	Version not specified; RRID:SCR_014239

### Experimental model and subject details

#### Human subjects

This study was approved by the Ethics Committee of the First Affiliated Hospital of Gannan Medical University (Approval No. 2022293). Written informed consent was obtained from all participating patients with active rheumatoid arthritis (RA). Bone marrow and peripheral blood samples were collected from RA patients undergoing bone marrow aspiration/biopsy or blood draws, respectively. Synovial tissue was obtained from an RA patient with a swollen knee joint. Patient demographics (e.g., age, sex) were not specified in the study but can be provided upon request. All procedures were conducted under sterile conditions with local anesthesia to minimize discomfort.

#### Animals

Female DBA/1 mice (8 weeks old, 18–20 g) were used to establish the collagen-induced arthritis (CIA) model. Mice were housed under specific pathogen-free conditions with a 12-hour light/dark cycle, controlled temperature (22 ± 2°C), and *ad libitum* access to food and water. Animal experiments were approved by the Ethics Committee of the First Affiliated Hospital of Gannan Medical University (Approval No. 2022293).

#### Cell lines

Normal human bone marrow-derived mesenchymal stem cells (BMSCs) were obtained from Procell Life Science & Technology Co., Ltd. (Wuhan, China; accession number: CVCL_A9JT). BMSCs were cultured in Dulbecco’s Modified Eagle Medium (DMEM) supplemented with 10% fetal bovine serum (FBS) and 1% penicillin-streptomycin at 37°C in a humidified 5% CO2 atmosphere. RA BMSCs were isolated from bone marrow aspirates of RA patients, and RA fibroblast-like synoviocytes (RA-FLS) were isolated from RA patient knee synovium, as described below.

### Method details

#### BMSC isolation and characterization

RA BMSCs were isolated from bone marrow aspirates of active RA patients, as described previously (ref.^34^). Briefly, bone marrow was diluted 1:1 with phosphate-buffered saline (PBS) and layered over Ficoll-Paque for density gradient centrifugation (400 × g, 30 min). The mononuclear cell layer was collected, washed thrice with PBS, and resuspended in DMEM with 10% FBS and 1% penicillin-streptomycin. Cells were cultured at 37°C with 5% CO2, with medium changes every 3–4 days until 70–80% confluence. BMSCs were characterized by flow cytometry using antibodies against CD29 (1:500), CD34 (1:20), CD44 (1:10), CD45 (1:500), CD90 (1:40), and CD105 (1:50) (Abcam, USA) on a BD flow cytometer.

#### BMSC-exosome isolation and identification

Exosomes were isolated from BMSC culture supernatant using the Hieff® Quick Exosome Isolation Kit (Yeasen, China) per the manufacturer’s instructions. Exosome protein content was quantified with a BCA Protein Assay Kit (Sangon, China). Particle size was analyzed using a Nanosight NS300 (Malvern, UK). Exosome markers (CD63, CD81, TSG101) were detected by western blot using specific antibodies (Affinity, China).

#### RA naïve CD4^+^ T cell isolation

Naïve CD4^+^ T cells were isolated from peripheral blood mononuclear cells of RA patients using the Human CD4 Naïve T Cell Isolation Kit (Stemcell, USA). Isolated cells were activated and expanded in T-cell Culture and Expansion Medium (Takara, Japan) at 37°C with 5% CO2.

#### Normal BMSC and RA naïve CD4^+^ T cell co-culture

Normal BMSCs were pre-treated with 10 μM GW4869 (Selleck, China) for 24 hours to inhibit exosome secretion (BMSC-GW4869). Normal BMSCs, BMSC-GW4869, or 100 ng of BMSC-derived exosomes (BMSC-Exo) were seeded at 5 × 10^4^ cells in the apical chamber of a transwell system. RA naïve CD4^+^ T cells were co-cultured in the basolateral chamber. CD4^+^ T cell differentiation was performed as described below, and Th17/Treg proportions and IL-17A/IL-10 concentrations in the supernatant were assessed by flow cytometry and ELISA, respectively.

#### RA naïve CD4^+^ T cell differentiation

RA naïve CD4^+^ T cells (1 × 10^5^) were cultured in 24-well plates for 3 days at 37°C in Th17 or Treg differentiation medium. Th17 medium contained 10 ng/mL TGF-β, 10 ng/mL IL-6, 10 ng/mL IL-1β, 10 ng/mL IL-23, anti-IL-4 (1:150), and anti-IFN-γ (1:100). Treg medium contained 100 U/mL IL-2 and 5 ng/mL TGF-β. Differentiation was assessed by flow cytometry for Th17 (IL-17A+) and Treg (FoxP3+) cells.

#### Normal BMSC lentiviral infection

Normal BMSCs were infected with lentiviruses for JKAP overexpression (oeJKAP), JKAP knockdown (shJKAP), or scramble control (Hanbio Biotechnology, China) at a multiplicity of infection (MOI) of 20 with 2 μg/mL polybrene (Beyotime, China). After 48 hours, JKAP expression was evaluated by RT-qPCR and western blot. Exosomes from infected BMSCs were isolated and analyzed for JKAP expression.

#### Exosome treatment of RA naïve CD4^+^ T cells

BMSC-derived exosomes (100 ng) were cultured with RA naïve CD4^+^ T cells for 48 hours at 37°C. JKAP, AKT, and ERK pathway expression was assessed by western blot. T cell differentiation was performed post-treatment, and Th17/Treg proportions and IL-17A/IL-10 levels were measured.

#### RA-FLS isolation and culture

RA-FLS were isolated from RA patient knee synovium, as described previously (ref.^35^). Synovial tissue was minced, digested, and cultured in DMEM with 10% FBS at 37°C with 5% CO2. Cells were identified morphologically using an inverted microscope (Motic, China).

#### RA-FLS and differentiated RA CD4^+^ T cell Co-culture

RA-FLS (4 × 10^5^) were seeded in plates, and Th17-differentiated RA CD4^+^ T cells were added in Th17 differentiation medium. RA-FLS proliferation was assessed at 0, 24, 48, and 72 hours using Cell Counting Kit-8 (GlpBio, USA). At 48 hours, apoptosis (TUNEL assay), invasion (Transwell assay), and supernatant cytokine levels (IL-6, IL-8, CCL2, MMP3) were evaluated.

#### RA naïve CD4^+^ T cell infection and inhibitor treatment

RA naïve CD4^+^ T cells were infected with shJKAP or scramble lentiviruses at MOI 20 with 6 μg/mL polybrene. Cells were treated with 10 nM MK2206 (AKT inhibitor) or PD98059 (ERK inhibitor) (Selleck, USA) for 48 hours. JKAP expression and AKT/ERK activation were assessed. T cell differentiation and RA-FLS co-culture were performed as described, with Th17/Treg proportions, cytokine levels, and RA-FLS proliferation/apoptosis/invasion evaluated.

#### RT-qPCR

RNA was extracted using the Total Exosome RNA & Protein Isolation Kit (Invitrogen, USA) or Trizol Reagent (Sangon, China). cDNA was synthesized from 500 ng RNA using the First Strand cDNA Synthesis Kit (Beyotime, China). qPCR was performed with SYBR Green qPCR Master Mix (Beyotime, China) on a 7500 Fast Real-Time PCR System (Applied Biosystems, USA) with the following conditions: 95°C for 2 min, then 40 cycles of 95°C for 15 s and 61°C for 30 s. Relative expression was calculated using the 2-ΔΔCt method with GAPDH as the internal control. Primer sequences are listed in the key resources table.

#### RA-FLS proliferation and apoptosis

RA-FLS proliferation was measured using Cell Counting Kit-8 (GlpBio, USA) after 2-hour incubation at 37°C, with optical density read at 450 nm. Apoptosis was assessed by TUNEL assay (Beyotime, China) after fixation and permeabilization, with imaging on an inverted fluorescence microscope (Motic, China).

#### Transwell invasion assay

RA-FLS (4 × 10^4^) were seeded in FBS-free medium in Matrigel® Invasion Inserts (Corning, USA). The lower chamber contained 10% FBS medium. After 24 hours at 37°C, invaded cells were fixed, stained with 0.1% crystal violet, and imaged using an inverted microscope.

#### Collagen-induced arthritis (CIA) model

CIA was induced in female DBA/1 mice by intradermal injection of chicken type II collagen emulsified in complete Freund’s adjuvant on day 0, followed by a booster on day 7. Sham mice received saline. On day 14, CIA mice were divided into four groups (n=6): CIA, CIA & Scramble-Exo, CIA & oeJKAP-Exo, and CIA & shJKAP-Exo. Exosomes from lentivirus-infected BMSCs were injected intravenously every 2 days. Arthritis index was evaluated weekly (ref.^36^). On day 49, mice were euthanized, and serum, spleen, synovial tissue, and synovial fluid were collected for ELISA, flow cytometry, HE staining, and IHC.

#### HE and IHC staining

Synovial tissue was fixed, paraffin-embedded, and sectioned (4 μm). HE staining was performed using the HE Stain Kit (Solarbio, China). For IHC, sections were deparaffinized, rehydrated, and subjected to antigen retrieval. Sections were incubated with JKAP antibody (1:200, Invitrogen, USA) overnight at 4°C, followed by HRP-linked secondary antibody (1:20000, Invitrogen, USA) for 1 hour at room temperature. Staining was developed with a DAB kit (Solarbio, China) and counterstained with hematoxylin. Images were captured using an inverted microscope.

#### ELISA

Supernatant, serum, and synovial fluid were analyzed for IL-17A, IL-10, IL-6, IL-8, CCL2, and MMP3 using commercial ELISA kits (Solarbio, China) per the manufacturer’s instructions.

#### Flow cytometry

Th17 and Treg cells were analyzed using Th17/Treg Phenotyping Kits (BD, USA). Cells were stimulated with 50 ng/mL PMA, 1 μg/mL ionomycin, and BD GolgiStop™ for 5 hours at 37°C, then fixed, permeabilized, and stained with antibody cocktails. Analysis was performed on a BD flow cytometer.

#### Western Blot

Cells were lysed in RIPA buffer with protease/phosphatase inhibitors (Beyotime, China). Proteins were quantified using a BCA kit (Sangon, China), separated on 4–20% precast gels (Invitrogen, USA), and transferred to nitrocellulose membranes (Solarbio, China). Membranes were blocked with 3% non-fat milk for 50 min at 37°C, incubated with primary antibodies overnight at 4°C, and then with secondary antibody (1:200000, Invitrogen, USA) for 1 hour at 37°C. Bands were detected using ECL reagent (Sangon, China) and analyzed with Image J 1.8 (NIH, USA).

### Quantification and statistical analysis

Data are presented as mean ± standard deviation from at least three independent experiments. Statistical analysis was performed using GraphPad Prism 7 (GraphPad, USA). Comparisons between two groups used unpaired t-tests, while multiple comparisons used Tukey’s test. Non-parametric tests were applied where appropriate. A p-value < 0.05 was considered statistically significant.

#### Statistical analysis

Data were analyzed using an unpaired t-test or Tukey’s multiple comparisons test and are presented as mean ± standard deviation. Data analysis and graph plotting were performed using GraphPad Prism 7 (GraphPad, USA). A p-value of less than 0.05 was considered statistically significant. All experiments were conducted in triplicate. Non-parametric test was used for this study.
