# Supplementary material for: BMSC exosomes deliver JKAP to restore Th17/Treg balance via AKT/ERK, alleviating rheumatoid arthritis
Source: iScience. 2025 Jun 6;28(7):112832. doi: 10.1016/j.isci.2025.112832 (PMC12271589; doi:10.1016/j.isci.2025.112832)

iScience, Volume *28*

Supplemental information

BMSC exosomes deliver JKAP to restore Th17/Treg balance via AKT/ERK, alleviating rheumatoid arthritis

Fang-Tian Xu, Yu Ling, Hui-Xian Wei, Lingzhang Meng, Dong Yin, Zhong-Hong Lai, Yu Huang, Xiao Huang, Hai-Ye Li, Qin-Wen Luo, Jian Song, Qiang Tang, and Hong-Mian Li

Supplementary Figure Legends

Supplementary Figure 1. Validation of BMSC (3rd generation) and its exosome. BMSC morphology (A) and surface marker levels (B). BMSC-exosome size distribution (C) and marker levels (D). JKAP expression in BMSC (E) and its exosome (F) between RA patients and controls. Data are represented as mean ± SEM. 5 samples in each group. Similar results were acquired from at leat 3 independent experiments.
***: $P < 0.001$.

Supplementary Figure 2. Confirmation of Genetic Modification of JKAP. Comparison of JKAP mRNA expression (A), western blot image examples (B), and quantified JKAP protein expression (C) in RA $CD4^+$ T cells among groups. Data are represented as mean ± SEM. 6 samples in each group. Similar results were acquired from at leat 3 independent experiments.
ns: no significance; *: $P < 0.05$; **: $P < 0.01$; ***: $P < 0.001$.

Supplementary Figure 3. The hypothesis diagram.

Supplementary Figure 1. Validation of BMSC and Its Exosome

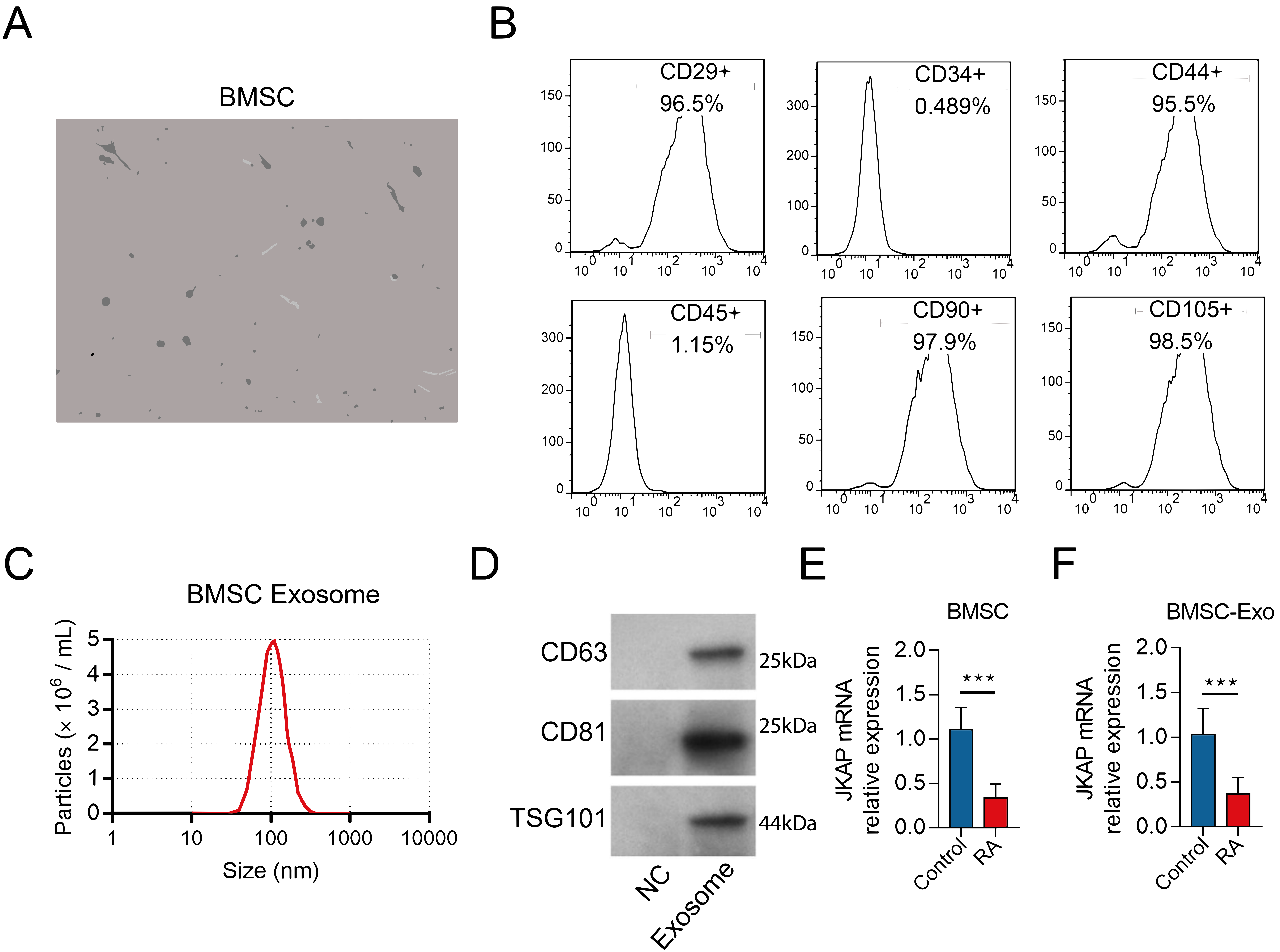

Supplementary Figure 2. Confirmation of Genetic Modification of JKAP

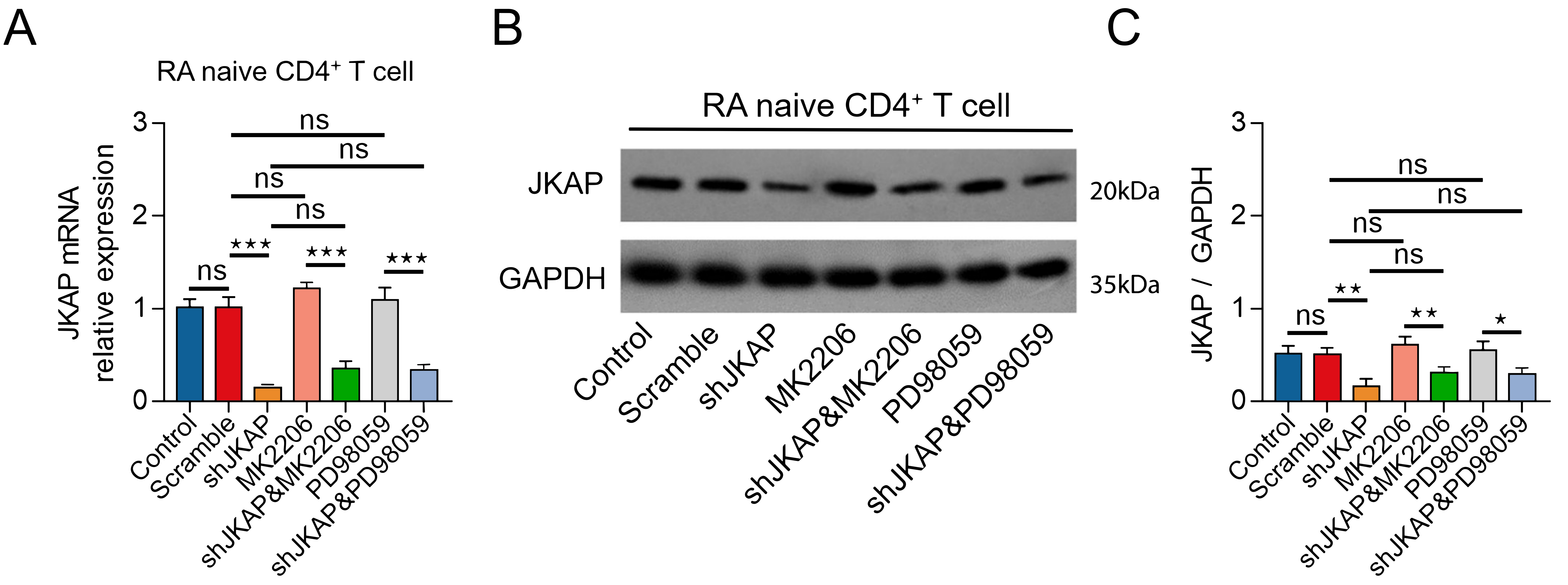

Supplementary Figure 3. The Hypothesis Diagram

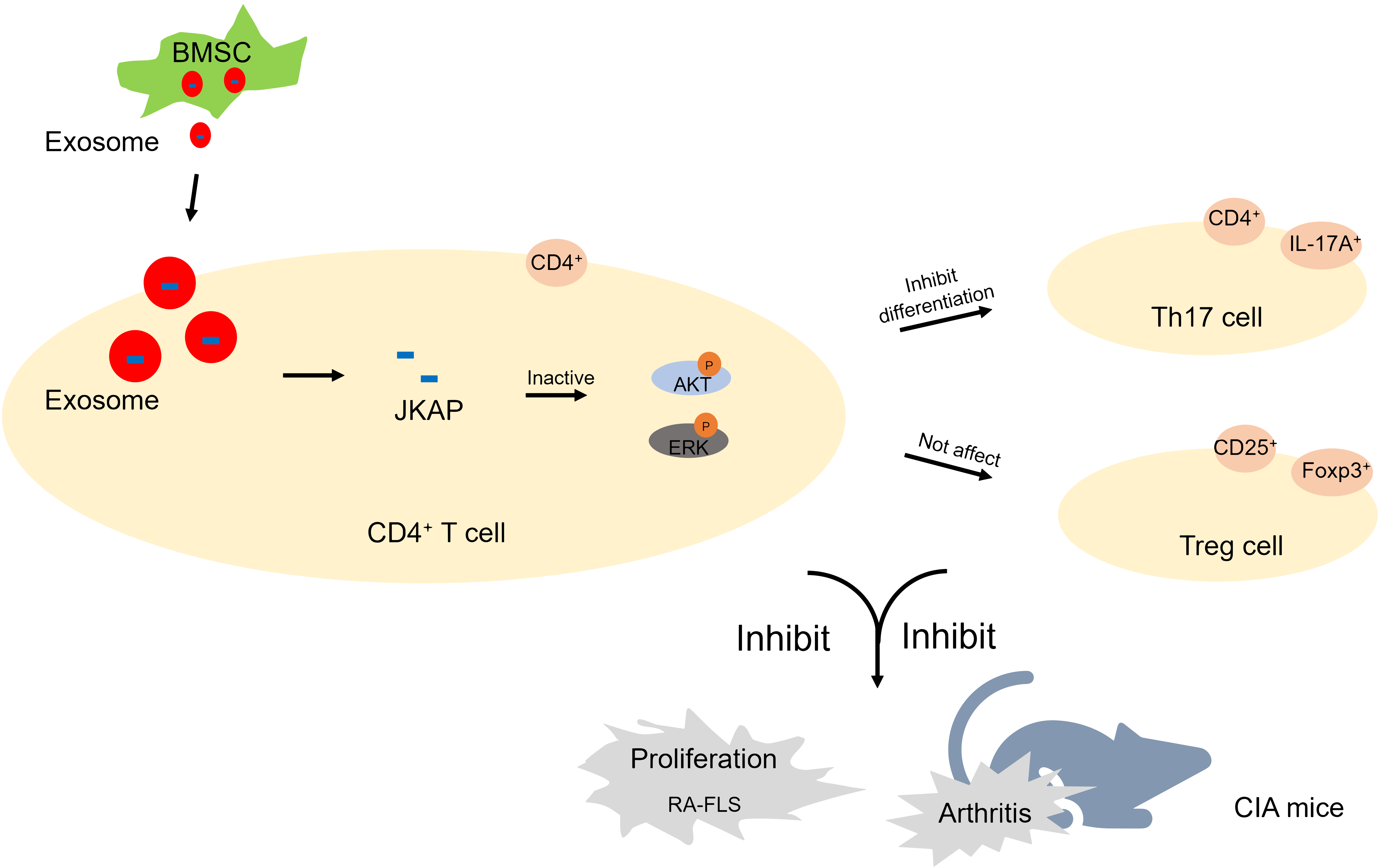

Supplement: Document S1. Figures S1–S3 [file mmc1.pdf]
